# Method for Film Thickness Mapping with an Astigmatic Optical Profilometer

**DOI:** 10.3390/s22082865

**Published:** 2022-04-08

**Authors:** Hsien-Shun Liao, Shih-Han Cheng, En-Te Hwu

**Affiliations:** 1 Department of Mechanical Engineering, National Taiwan University, Taipei 10617, Taiwan; r07522638@ntu.edu.tw; 2 Department of Health Technology, Technical University of Denmark, 2800 Lyngby, Denmark; etehw@dtu.dk

**Keywords:** astigmatism, optical profilometer, thickness measurement

## Abstract

An astigmatic optical profilometer is a precision instrument with advantages such as high resolution, high bandwidth, a compact size, and low cost. However, current astigmatic optical profilometers measure only surface morphology, and their potential for capturing subsurface information remains underutilized. In this study, we developed a method for measuring the thickness of transparent thin films with an astigmatic optical profilometer. Experimental results demonstrate that the thickness of transparent films tens of micrometers thick can be accurately measured. The maximum thickness measurable through our system is approximately 100 μm, which may be increased to 1.2 mm through the use of a scanner with a greater travel range. A coupling problem occurs for films <25 μm in thickness. However, to solve this problem, we devised a decoupling method, which was experimentally implemented to successfully measure a 18-μm-thick film. Moreover, the ability to obtain 3D images, including of both the upper and lower surfaces, was demonstrated.

## 1. Introduction

Thin membrane structures are widely found in nature, and many of them, such as the corneal tear film, cell membranes, and skin corneocytes, play crucial roles in the human body [1,2,3,4,5]. Thin films are used in various industrial applications, such as water treatment, solar cells, optical filters, and biosensors [6,7,8,9,10,11], and thickness measurement is a crucial technique for examining the properties of manufactured films.

Various methods, including stylus profilometry, atomic force microscopy (AFM), interferometry, and ellipsometry, have been utilized to measure film thickness [12,13,14]. By using a sharp probe to scan a measured surface, contact-based methods, including stylus profilometry and AFM, can provide high-resolution images that can identify membranes only a single atom thick on a substrate [15]. In these methods, the probe must contact both the film and substrate surface to measure the film’s thickness. Therefore, a major limitation of contact methods is that the associated substrate must be at least partially exposed. By contrast, noncontact methods are usually based on optical principles and are often more expedient than contact methods are. Moreover, noncontact methods avoid the damage to the sample that results from the interaction force between the sample and probe, as occurs with contact methods. The appropriate measurement method depends on the requirements of the application [16]. For instance, spectroscopic ellipsometry (SE) is commonly used for measuring transparent films with thicknesses ranging from nanometers to tens of micrometers [17,18,19]. However, model-based SE thickness calculations require precise modeling and parameter selection to obtain accurate results [20,21,22].

Astigmatic optical profilometers based on digital versatile disk (DVD) heads offer the advantages of high resolution, a compact size, high bandwidth, and low cost [23,24]. By utilizing the astigmatic method, the astigmatic optical profilometers can avoid the multiple reflections problem occurred in conventional interferometry [25]. Moreover, the astigmatic optical profilometers can also provide surface image with microscale lateral resolution, which is not available in conventional ellipsometry with a millimeter beam size [26]. We previously developed a *z*-axis modulation method to decouple surface morphology and reflectivity, enabling the quantitative height measurement of surfaces consisting of complex materials [27]. In [28], we developed a resonant *z*-axis scanner to improve the imaging rate of astigmatic optical profilometers. In the present study, we developed a novel method for film thickness measurement using an astigmatic optical profilometer.

## 2. Materials and Methods

### 2.1. Thickness Calculation Method

**Figure 1:**
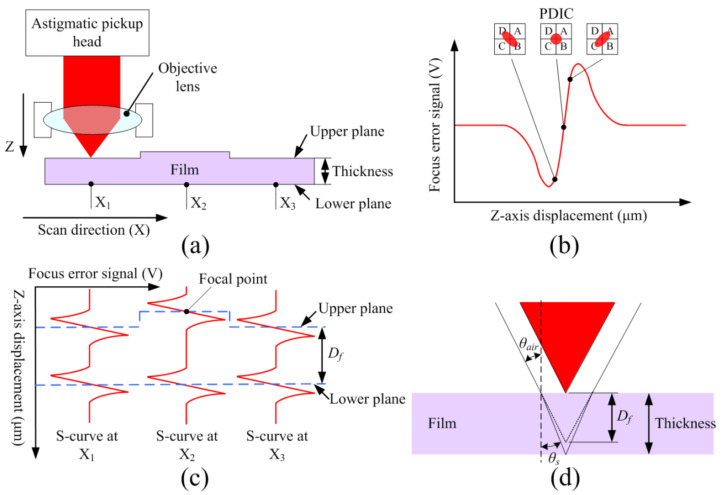
(**a**) Illustration of thickness measurement along the scanning direction. (**b**) Typical S-curve feature on a surface. (**c**) FES vs. *z*-axis displacement curves at corresponding positions in (**a**). (**d**) Illustration of refraction calibration.

Figure 1a illustrates the proposed method for the measurement of the thickness of transparent films. A laser from an astigmatic pickup head is focused on a sample surface, and the laser reflection is received by a photodetector integrated chip (PDIC). The PDIC consists of four quadrant photosensors (A, B, C, D), which generate voltage signals (*S_A_*, *S_B_*, *S_C_* and *S_D_*) through an amplifier circuit. Due to the astigmatic effect of the pickup head, the *z*-axis displacement of the sample causes shape variation of the laser spot on the PDIC. This shape variation can be detected by a focus error signal (FES) defined as (*S_A_* + *S_C_*) − (*S_B_* + *S_D_*). The relation between the FES and *z*-axis displacement between the pickup head and sample creates an S-curve feature around the laser’s focal position as illustrated in Figure 1b. In common profilometer mode, the vertical position of the pickup head is adjusted to position the FES within the linear region of the S-curve; the FES can then represent the surface profile as the laser scans the surface. In the thickness measurement mode, the objective lens or the measured sample is moved vertically to obtain the FES—*z*-axis displacement curve for each image pixel, as shown in Figure 1c. Two S-curve features are generated by the laser reflection from the upper and lower planes. Through the calculation of the *z*-axis positions of the upper and lower focal points, the upper and lower surface profiles can be obtained, as indicated in Figure 1c. Moreover, the vertical displacement *D_f_* between upper and lower focal points can also be calculated. To obtain the film thickness, the refraction effect in the film sample needs to be considered, as illustrated in Figure 1d. The film thickness can be calculated as in (1):

(1) $$\mathrm{Thickness}\;=\;D_{f}\frac{\tan\theta_{air}}{\tan\theta_{s}},$$

where *θ_air_* and *θ_s_* represent the incidence angle and the refraction angle, respectively. In our system, the incidence angle *θ_air_* equals to 36.87°, which can be calculated as in (2):

(2) $$\mathrm{NA}\;=\;n_{air}\sin\theta_{air},$$

where the numerical aperture (NA) of the objective lens is 0.6, and the refractive index is approximately 1 in air. The refraction angle *θ_s_* in the film sample can be calculated as in (3):

(3) $$n_{air}\sin\theta_{air}\;=\;n_{s}\sin\theta_{s},$$

where the sample refractive index *n_s_* is required to calculate the refraction angle *θ_s_*. Through scanning of the *xy* plane, 3D images of the upper and lower surfaces can be obtained, and a spatial thickness distribution can be determined.

### 2.2. Decoupling of S-Curves

**Figure 2:**
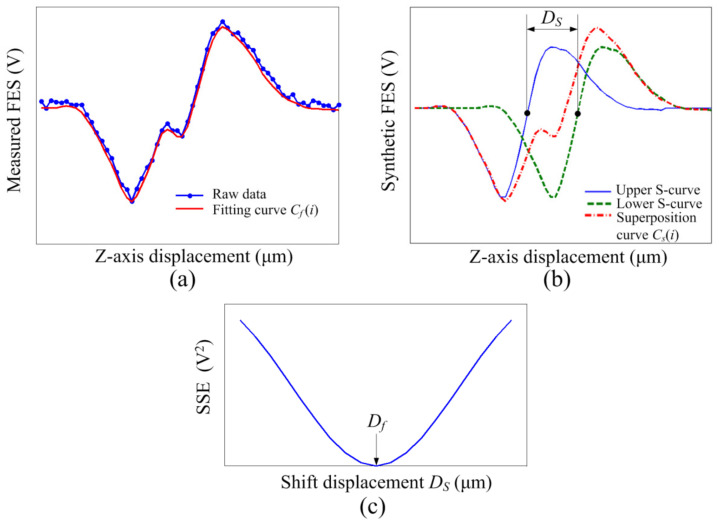
(**a**) FES vs. *z*-axis displacement curve illustrating coupling problem. (**b**) Synthetic curve from two S-curves. (**c**) Sum of squared error vs. *D_s_* curve.

One limitation of film thickness measurement is the coupling of the upper and lower S-curves, which occurs for films <25 μm thick according to the S-curve range of the DVD pickup head. In this study, we proposed a decoupling method for our astigmatic optical profilometer. Figure 2a shows an FES—*z*-axis displacement curve exhibiting the aforementioned coupling problem. In our method, a fitting curve *C_f_*(*i*) based on raw data is calculated to obtain an FES for an arbitrary *z*-axis displacement and eliminate noise. The *C_f_*(*i*) was synthesized by eight sinusoidal functions using Matlab software. A pure S-curve feature without the coupling issue is required; this can be obtained by measuring a thicker film composed of the same material. Through the generation of two S-curves with a displacement shift *D_s_*, a synthetic FES—*z*-axis displacement curve *C_s_*(*i*) can be calculated according to the superposition principle, as illustrated in Figure 2b. Subsequently, the sum of squared error (SSE) at different *D_s_* can be calculated as in (4):

(4) $$\mathrm{SSE}\;=\;\sum_{i\;=\;1}^{n}{\left(C_{f}\left(i\right)\;-\;C_{s}\left(i\right)\right)}^{2},$$

where *i* and *n* indicate the data index and number of data, respectively. Figure 2c displays the relation between SSE and *D_s_*; the *D_s_* where SSE is minimized indicates the *D_f_* for calculating the film thickness.

### 2.3. Astigmatic Optical Profilometer

**Figure 3:**
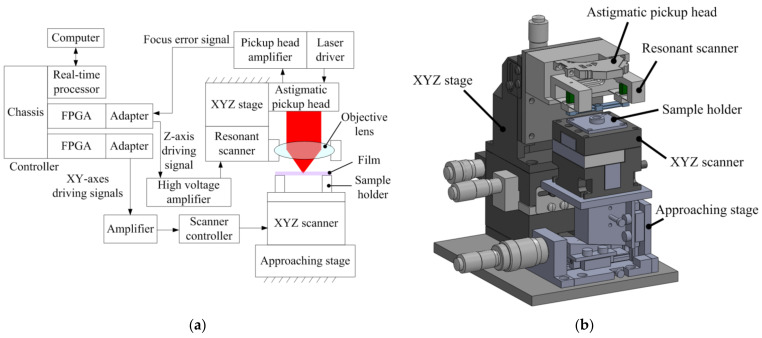
(**a**) System configuration and (**b**) mechanical design of astigmatic optical profilometer.

We used the previously designed astigmatic optical profilometer featuring a resonant scanner for high-speed *z*-axis modulation [28]. The system configuration and mechanical design of the astigmatic optical profilometer are presented in Figure 3a,b, respectively. To measure the S-curve for each image pixel, the resonant scanner contains the objective lens of an astigmatic pickup head (TOP1100s, TopRay Technologies, Hsinchu, Taiwan) and performs vertical sinusoidal movements. The resonant scanner has a resonant frequency of 1.6 kHz and a travel range of >87 μm. A film sample is placed on a tubular sample holder to avoid light reflected by the holder’s surface. An XYZ stage (XC2-60SMW and ZA-60MBW, Tsukumo Engineering, Sayama, Japan) is used to move the pickup head into its measuring range and to adjust its position. The FES generated from a customized amplifier (high-speed TopRay driver v122, Strømlinet Nano) is acquired by a programmable controller (PXIe system, National Instruments, Austin, TX, USA) equipped with a chassis (PXIe-1062Q), a real-time controller (PXIe-8840), and two field-programmable gate array modules (FPGAs) (PXIe-7961R) with adaptors (NI-5781). The controller directs the vertical movement of the resonant scanner and the XY scanning of a closed-loop XYZ piezoelectric scanner (P-611.3S NanoCube, Physik Instrumente, Karlsruhe, Germany) through a high-voltage amplifier (PD200, PiezoDrive) and a scanner controller (E-664.S3 Piezo Controller, Physik Instrumente, Karlsruhe, Germany), respectively. The *z*-axis displacement of the XYZ scanner has a range of 100 μm, which can also be used for single-point thickness measurement. For 3D imaging, the resonant scanner is essential for reducing measurement time.

## 3. Results and Discussion

### 3.1. Single-Point Film Thickness Measurement

**Figure 4:**
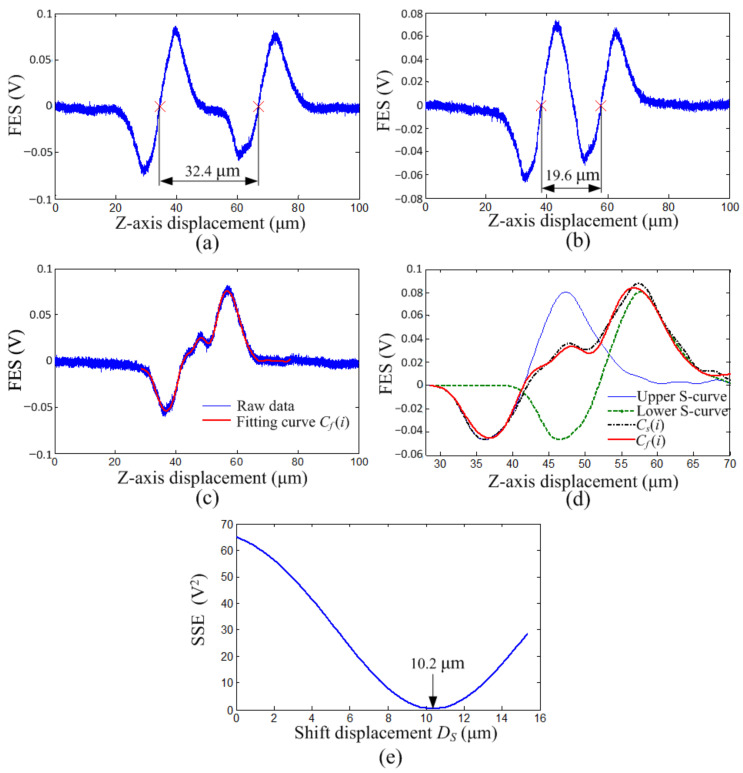
FES vs. *z*-axis displacement curve for OPP films thicknesses of (**a**) 55, (**b**) 33, and (**c**) 18 μm. (**d**) Synthetic curves with *D_s_* of 10.2 μm. (**e**) SSE vs. *D_s_* curve.

To examine our single-point thickness measurement, we measured three biaxially oriented polypropylene (OPP) films (Uni Film) with thicknesses of 55, 33, and 18 μm, which were validated by an optical microscope (IX73, Olympus). The refractive index of the OPP film was 1.49, which was provided by the manufacturer. According to (3), the corresponding refraction angle *θ_s_* was 23.75°. The OPP films were moved vertically by the XYZ scanner, and the FES—*z*-axis displacement curves for the 55, 33, and 18 μm OPP films are displayed in Figure 4a–c, respectively. The focal points on 55-μm-thick and 33-μm-thick films were identified through linear fitting in the linear regions of the S-curves; the corresponding *D_f_* were 32.4 and 19.6 μm, respectively. By applying (1), the corresponding thicknesses of 55.2 μm and 33.4 μm were calculated, which were consistent with the results of 53.2 μm and 32.7 μm obtained by a 3D laser scanning confocal microscope (VK-X, KEYENCE) adopting the same refraction parameters. The thickness of the 18 μm-thick film was determined through the S-curve decoupling method due to the aforementioned coupling problem. The fitting curve *C_f_*(*i*) of the raw data was obtained from the sum of eight sinusoidal functions, indicated by the solid red lines in Figure 4c,d. Through the same fitting method, a pure S-curve feature was obtained from the lower S-curve feature shown in Figure 4a and used to synthesize the superposition curve *C_s_*(*i*) (black dotted–dashed line) displayed in Figure 4d. The SSE between *C_f_*(*i*) and *C_s_*(*i*) at different *D_s_* values is displayed in Figure 4e, which indicates that the best fit occurred at *D_s_* of 10.2 μm. Figure 4d displays the synthetic result for *D_s_* of 10.2 μm, indicating that an appropriate *C_s_*(*i*) was obtained. The corresponding thickness calculated by using (1) was 17.4 μm, which was also consistent with the result of 16.5 μm obtained by the scanning confocal microscope.

### 3.2. 3D Imaging

**Figure 5:**
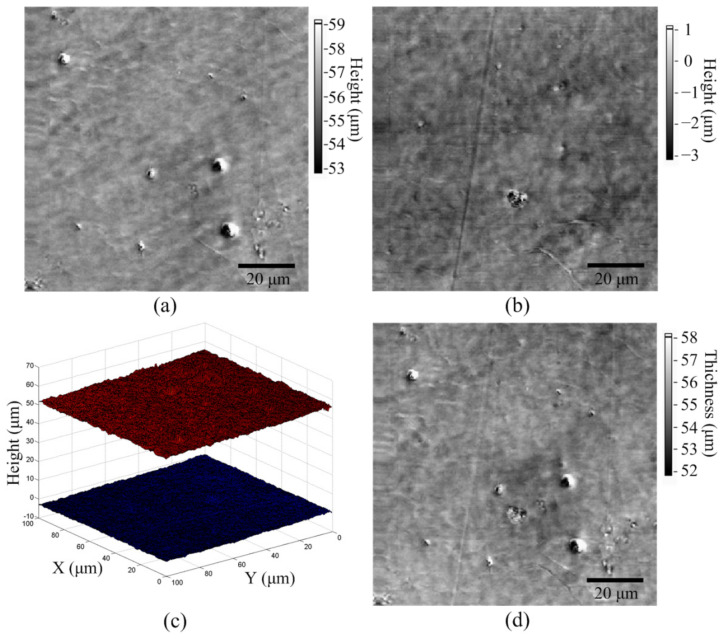
Height images of (**a**) upper and (**b**) lower surfaces. (**c**) 3D image of top and bottom surfaces. (**d**) Thickness image.

We employed the 55-μm-thick OPP film to test the 3D imaging function. To improve imaging speed, the resonant scanner was used to move the objective lens vertically. The resonant scanner was driven at a frequency of 1.57 kHz and had a travel range of 67.6 μm. An area of 100 μm × 100 μm was scanned with an image resolution of 256 pixels × 256 pixels at a scan rate of 1 line/s. The *z*-axis positions of the upper and lower focal points for each pixel were calculated through the linear fitting method. The images of the upper and lower surfaces are shown in Figure 5a,b, and a 3D image of both surfaces is displayed in Figure 5c. The images reveal various defects, such as particles and scratches, on the upper and lower surfaces, and no image coupling can be observed. The thickness image is displayed in Figure 5d, and the average thickness from all 65 536 pixels was 55.2 ± 0.6 μm (mean ± standard deviation). 

## 4. Conclusions

In this study, an astigmatic optical profilometer with a thickness measuring function was created. Experimental results demonstrate that thicknesses of OPP films 18–55 μm thick can be measured accurately. Moreover, 3D images, including those of the upper and lower surfaces of films, can be obtained. Ideally, the maximum measurable thickness is limited by the working distance of the objective lens of the pickup head, which is approximately 1.28 mm for common DVD pickup. However, the *z*-axis travel range of the XYZ scanner is only 100 μm, limiting the maximum measurable thickness in our current system. The actually measurable thickness also depends on the sample refractive index as explained in the calculation method. On the other hand, S-curve coupling issue occurs for films <25 μm thick. By using our proposed decoupling method, we accurately measured a 18-μm-thick film. However, this method requires a known S-curve feature from the same material for estimating the superposition curve. In addition, differences between the synthetic and measured curves may cause additional measurement errors. The maximum thickness without S-curve coupling can be further reduced through the use of a Blu-ray pickup head, which has a shorter S-curve range of approximately 10 μm comparing with the red-light pickup head [29]. Moreover, the short-wavelength and the high-NA objective lens of the Blu-ray pickup head can also improve the spatial resolution of the 3D imaging. However, the maximum measurable thickness is reduced due to the short working distance of the Blu-ray pickup head. Furthermore, the thickness calculation was executed off-line using Matlab software currently. For the industrial applications, the calculation algorithm needs to be implanted into the FPGA for real-time thickness imaging in the future.

## References

[1] Branchet M.C., Boisnic S., Frances C., Robert A.M. (1990). Skin thickness changes in normal aging skin. Gerontology.

[2] King-Smith P.E., Fink B.A., Hill R.M., Koelling K.W., Tiffany J.M. (2004). The thickness of the tear film. Curr. Eye Res..

[3] Cwiklik L. (2016). Tear film lipid layer: A molecular level view. Biochim. Biophys. Acta.

[4] Wen P.J., Grenklo S., Arpino G., Tan X., Liao H.S., Heureaux J., Peng S.Y., Chiang H.C., Hamid E., Zhao W.D. (2016). Actin dynamics provides membrane tension to merge fusing vesicles into the plasma membrane. Nat. Commun..

[5] Chan T.C., Ye C., Ng P.K., Li E.Y., Yuen H.K., Jhanji V. (2015). Change in Tear Film Lipid Layer Thickness, Corneal Thickness, Volume and Topography after Superficial Cauterization for Conjunctivochalasis. Sci. Rep..

[6] Pendergast M.M., Hoek E.M.V. (2011). A review of water treatment membrane nanotechnologies. Energy Environ. Sci..

[7] Sengupta D., Das P., Mondal B., Mukherjee K. (2016). Effects of doping, morphology and film-thickness of photo-anode materials for dye sensitized solar cell application—A review. Renew Sustain. Energ. Rev..

[8] Liu C., Zhang Q., Wang D., Zhao G., Cai X., Li L., Ding H., Zhang K., Wang H., Kong D. (2018). High Performance, Biocompatible Dielectric Thin-Film Optical Filters Integrated with Flexible Substrates and Microscale Optoelectronic Devices. Adv. Optical Mater..

[9] Pan Y., Liu H., Zheng Z. (2021). Design and fabrication of optical filters for infrared imaging temperature measurement. Optik.

[10] Kottapalli A.G.P., Tan C.W., Olfatnia M., Miao J.M., Barbastathis G., Triantafyllou M. (2011). A liquid crystal polymer membrane MEMS sensor for flow rate and flow direction sensing applications. J. Micromech. Microeng..

[11] Wang D., Ba D., Hao Z., Li Y., Sun F., Liu K., Du G., Mei Q. (2018). A novel approach for PDMS thin films production towards application as substrate for flexible biosensors. Mater. Lett..

[12] Piegari A., Masetti E. (1985). Thin film thickness measurement: A comparison of various techniques. Thin Solid Films.

[13] Benoit M., Bataillon C., Gwinner B., Miserque F., Orazem M.E., Sánchez-Sánchez C.M., Tribollet B., Vivier V. (2016). Comparison of different methods for measuring the passive film thickness on metals. Electrochim. Acta.

[14] Giurlani W., Berretti E., Innocenti M., Lavacchi A. (2020). Measuring the Thickness of Metal Coatings: A Review of the Methods. Coatings.

[15] Shearer C.J., Slattery A.D., Stapleton A.J., Shapter J.G., Gibson C.T. (2016). Accurate thickness measurement of graphene. Nanotechnology.

[16] Giurlani W., Berretti E., Lavacchi A., Innocenti M. (2020). Measuring the Thickness of Metal Films: A Selection Guide to the Most Suitable Technique. Mater. Proc..

[17] Isić G., Jakovljevic M., Filipovic M., Jovanovic D.M., Vasic B., Lazovic S., Puac N., Petrovic Z.L., Kostic R., Gajic R. (2011). Spectroscopic ellipsometry of few-layer graphene. J. Nanophotonics.

[18] Garriga M., Alonso M.I., Dominguez C. (1999). Ellipsometry on very thick multilayer structures. Phys. Stat. Sol..

[19] Fodor B., Agocs E., Bardet B., Defforge T., Cayrel F., Alquier D., Fried M., Gautier G., Petrik P. (2016). Porosity and thickness characterization of porous Si and oxidized porous Si layers—An ultraviolet–visible–mid infrared ellipsometry study. Microporous Mesoporous Mater..

[20] Rosu D.-M., Ortel E., Hodoroaba V.-D., Kraehnert R., Hertwig A. (2017). Ellipsometric porosimetry on pore-controlled TiO2 layers. Appl. Surf. Sci..

[21] Secondo R., Fomra D., Izyumskaya N., Avrutin V., Hilfiker J.N., Martin A., Özgür Ü., Kinsey N. (2019). Reliable modeling of ultrathin alternative plasmonic materials using spectroscopic ellipsometry [Invited]. Opt. Mater. Express.

[22] Li H., Cui C., Xu X., Bian S., Ngaojampa C., Ruankham P., Jaroenjittchai A.P. (2020). A review of characterization of perovskite film in solar cells by spectroscopic ellipsometry. Sol. Energy.

[23] Hwu E.T., Hung S.K., Yang C.W., Huang K.Y., Hwang I.S. (2008). Real-time detection of linear and angular displacements with a modified DVD optical head. Nanotechnology.

[24] Hwu E.T., Illers H., Jusko L., Danzebrink H.U. (2009). A hybrid scanning probe microscope (SPM) module based on a DVD optical head. Meas. Sci. Technol..

[25] Kim S.-W., Kim G.-H. (1999). Thickness-profile measurement of transparent thin-film layers by white-light scanning interferometry. Appl. Opt..

[26] An I. (2015). Application of imaging ellipsometry to the detection of latent fingermarks. Forensic Sci. Int..

[27] Liao H.-S., Huang G.-T., Tu H.-D., Lin T.-H., Hwu E.-T. (2018). A novel method for quantitative height measurement based on an astigmatic optical profilometer. Meas. Sci. Technol..

[28] Liao H.-S., Cheng S.-H., Hwu E.-T. (2021). Development of a Resonant Scanner to Improve the Imaging Rate of Astigmatic Optical Profilometers. IEEE/ASME Trans. Mechatron..

[29] Sun W.-S., Lin Y.-N., Tien C.-L., Tsuei C.-H., Kuo C.-C., Chang J.-Y. (2012). Compact design for a unitary photo detector and single-path combo optical pickup head for Blu-ray disc, digital versatile disc and compact disc systems. Opt. Lasers Eng..

